# Kaempferol and Fisetin-Related Signaling Pathways Induce Apoptosis in Head and Neck Cancer Cells

**DOI:** 10.3390/cells12121568

**Published:** 2023-06-06

**Authors:** Robert Kubina, Kamil Krzykawski, Arkadiusz Dziedzic, Agata Kabała-Dzik

**Affiliations:** 1 Department of Pathology, Faculty of Pharmaceutical Sciences in Sosnowiec, Medical University of Silesia, 30 Ostrogórska Str., 41-200 Sosnowiec, Poland; adzik@sum.edu.pl; 2 Silesia LabMed, Centre for Research and Implementation, Medical University of Silesia in Katowice, 18 Medyków Str., 40-752 Katowice, Poland; kamil.krzykawski@sum.edu.pl; 3 Department of Conservative Dentistry with Endodontics, Medical University of Silesia, 40-055 Katowice, Poland; adziedzic@sum.edu.pl

**Keywords:** fisetin, kaempferol, apoptosis, head and neck cancer, cell cycle, caspase-3, cytochrome c

## Abstract

Despite the relative effectiveness of standard cancer treatment strategies, head and neck cancer (HNC) is still considered one of the leading causes of mortality and morbidity. While selected bioactive compounds of plant origin reveal a pro-apoptotic effect, kaempferol and fisetin flavonols have been reported as potential anti-cancer agents against malignant neoplasms. To date, their exact role in signaling pathways of head and neck cancer cells is largely unknown. Based on the various methods of cytotoxicity testing, we elucidated that kaempferol and fisetin inhibit proliferation, reduce the capacity of cell migration, and induce apoptosis in SCC-9, SCC-25, and A-253 HNC cells in a dose-dependent manner in vitro (*p* < 0.05, fisetin IC_50_ values of 38.85 µM, 62.34 µM, and 49.21 µM, and 45.03 µM, 49.90 µM, and 47.49 µM for kaempferol–SCC-9, SCC-25, and A-253, respectively). The obtained results showed that exposure to kaempferol and fisetin reduces Bcl-2 protein expression, simultaneously leading to the arrest in the G2/M and S phases of the cell cycle. Kaempferol and fisetin inhibit cell proliferation by interfering with the cell cycle, which is strongly associated with the induction of G2/M arrest, and induce apoptosis by activating caspase-3 and releasing cytochrome c in human HNC cells. In addition, investigating flavonols, by inhibiting anti-apoptotic proteins from the Bcl-2 family and damaging the mitochondrial transmembrane potential, increased the level of cytochrome c. While flavonols selectively induce apoptosis of head and neck cancer cells, they may support oncological therapy as promising agents. The discovery of new derivatives may be a breakthrough in the search for effective chemotherapeutic agents with less toxicity and thus fewer side effects.

## 1. Introduction

From the epidemiological perspective, malignant neoplasms are the second most common cause of mortality in the world. A substantial number of diagnosed cancers, among others, followed the civilizational changes that have occurred over the recent decades. The extension of the average life expectancy is deemed one of the reasons for the increase in cancer incidence, and it could be associated with exposure to carcinogens that contribute to the development of cancerogenous conditions [1]. Globocan 2020 data reported nearly 5 million new cancer cases and over 2 million cancer-related deaths in the European region according to the World Health Organization [2].

The head and neck cancers (HNCs) usually originate from the squamous cells that line the mucosal surfaces of the head and neck areas; thus, they are called head and neck squamous cell cancers (HNSCCs). HNCs may also originate from the salivary glands, sinuses, muscles, and nerves, although they are much less prevalent than squamous cell cancers [3]. The primary locations of HNCs are: the mouth, including the lips, the front two-thirds of the tongue, the gums, the lining inside the cheeks and lips, the floor of the mouth under the tongue, the hard palate, and a retromolar area of gingivae behind the wisdom teeth; the throat; and the larynx, paranasal sinuses, nasal cavity, and salivary glands [4].

Despite global scientific and clinical efforts, the progress in diagnostics and therapy that has been achieved in recent years has not resulted in a significant improvement in treatment outcomes in patients diagnosed with HNCs. Early diagnosis remains a key factor in determining the final prognosis of the patient due to limited therapeutic options [5]. In patients diagnosed with early stages of clinical advancement, standard therapeutic methods are sufficient to support oncological treatment; however, in other cases, the results are not satisfactory and are often associated with a high risk of side effects [5,6]. Supportive treatment, as a pivotal part of cancer therapy to reduce complications, is still not optimized.

Therapeutic toxicity and resistance to standard chemotherapeutic therapy are major clinical challenges in the management of HNCs. Although oncological advances have been implemented recently, the 5-year survival rate of HNC patients has not been substantially improved in recent decades. Currently, the increasing utilization of immunotherapy in cancer management may enhance clinical outcomes [5].

The approach to cancer prevention using new, non (minimally)-toxic agents of plant origin is indicated by researchers, focusing on flavonoids, which are commonly found in most plants and have a wide range of biological activities, such as antioxidative [7], anti-proliferative [8], anti-inflammatory [9], antibacterial/antiviral [10,11], as well as stimulating the immune system. These compounds are classified as plant secondary metabolites and have a significant impact on the biological system [12]. Widely occurring flavonols are one of the groups of flavonoids that are abundant in various types of nuts, wine, teas, and fruits and vegetables such as strawberries, apples, persimmons, onions, grapes, kiwi, peaches, cucumbers, and tomatoes [13]. Due to anti-angiogenic, anti-inflammatory, anti-cancer, antioxidant, and/or neuroprotective properties, they are considered as seno-therapeutic agents [13,14,15,16].

These compounds have a common structure consisting of two aromatic rings linked to three carbon atoms. It is generally believed that the number of hydroxyl substituents correlates with antioxidant activity. The correlation between the structure and other functions of flavonoids is not fully understood [17].

Fisetin (3,7,3′,4′-tetrahydroxyflavone) is a naturally occurring flavonol. The concentration of fisetin varies in different types of fruits and vegetables [18], while strawberries, apples, and persimmons contain the highest concentration. Fisetin might play a role in the treatment of diseases by modulating various biological pathways. Studies have shown the role of fisetin as an antioxidant [19] and an anti-inflammatory agent [20,21] in attenuating diabetic cardiomyopathy associated with oxidative stress, inflammation, and apoptosis [18]. Reportedly, its role in various types of cancer has been described. It exerts a disease-preventing effect by improving antioxidant status, inhibiting inflammation, inducing apoptosis, and inhibiting angiogenesis, as well as inhibiting growth factor production, which modulates other genetic pathways [22,23].

Kaempferol (3,4′,5,7-tetrahydroxyflavone), also known as indigo yellow, is a nutrient that is widely present in many plants and fruits and vegetables, such as tea [24], cabbage, broccoli, apples, grapes [25], citrus fruits, strawberries, beans, tomatoes, and onions [26]. The biological activity of polyphenols is largely due to their molecular shape and the relative spatial orientation of phenolic rings and hydroxyl groups [27]. The chemopreventive properties of kaempferol are largely attributed to its ability to induce apoptosis [28]. Kaempferol has also been found to increase the ability of chemotherapeutic agents to induce cell death, leading to the suggestion that these flavonoids may be useful as adjuvant chemotherapeutic agents that “sensitize” cancer cells to the anti-cancer effects of the main chemotherapeutic agent [29]. Apoptosis of cancer cells induced by anti-cancer drugs is one of the optimal anti-cancer strategies to underpin novel therapeutic methods [30].

Considering the urgent need to develop innovative modalities to support HNC therapy, the primary aim of this study is to investigate in vitro the multidirectional effects of fisetin and kaempferol toward three HNC reference cell lines, including the squamous cell cancer line (tongue) and the submandibular gland cancer line. Here, our study assessed comprehensively the impact of exposure to fisetin and kaempferol on cell growth inhibition, migration inhibition, apoptosis induction, changes in caspase-3 enzyme activity, the release of cytochrome c from mitochondria, mitochondrial membrane disruption, and cell cycle distribution.

## 2. Materials and Methods

### 2.1. Materials

Selected flavonols, including fisetin (cat. no. S2298, purity: 97.76%) and kaempferol (cat. no. 60010, purity: >97%), were purchased from Sigma-Chemical (St. Louis, MO, USA). In addition, sterile dimethyl sulfoxide (DMSO cat. no. D2650) and hydrocortisone (cat. no. H6909) purchased from Merc were used for the study. Cell culture media, fetal serum, antibiotics, PBS, and trypsin were manufactured by Corning (Corning Life Science, Painted Post, NY, USA).

### 2.2. Cell Culture

Three human HNC cell lines were used in this study, including human oral (tongue) squamous cell cancer lines SCC-9 and SCC-25 and submandibular gland cancer A-253. Human normal epithelial cells of the Beas-2B line were used as a control. The HNC lines were from the ATCC collection, and the correct cells were from the ECACC collection.

Tongue cancer cells were cultured according to the manufacturer’s instructions in DMEM/F-12 medium (1:1 ratio) supplemented with 400 ng/mL hydrocortisone. A-253 cells were cultured in McCoy’s 5a medium modified without the addition of hydrocortisone. The media were additionally supplemented with antibiotics (100 U/mL penicillin and 100 U/mL streptomycin) and 10% fetal bovine serum and cultured at 37 °C in a 5% CO_2_ atmosphere. Beas-2B cells were cultured in DMEM/F-12 medium (1:1 ratio) with the addition of non-inactivated fetal serum and antibiotics at a concentration of 100 U/mL penicillin and 100 U/mL streptomycin.

### 2.3. Fisetin and Kaempferol Treatment

A stock solution of fisetin and kaempferol (5 mg/mL) was prepared by dissolving the test compounds in DMSO and then stored frozen at −80 °C until use, but no longer than 14 days. Various concentrations from 5 to 100 mM were used for the tests. An additional control consisted of cells grown in a medium containing an equivalent amount of DMSO without the addition of flavonols. Studies confirm that DMSO may be safely used in in vitro studies using cell lines in a range from 0.6% to 0.15% [31].

### 2.4. Cell Proliferation Assay (WST-1 Test)

Briefly, cells were seeded in 96-well plates at 5 × 10^3^ cells/well in 100 μL of culture medium and allowed to grow logarithmically for 24 h. The medium was then replaced with medium supplemented with various concentrations of flavonols. Cells were incubated for 24, 48, and 72 h at 37 °C and 5% CO_2_. After this time, 10 µL of Cell Proliferation Reagent WST-1 (Cat. No. CELLPRO-RO, Roche, Mannheim, Germany) was added to each well and incubated for 4 h. The contents of the wells were then thoroughly mixed on a shaker for 1 min and the absorbance of the samples was measured against a background control as a blank test using a Varioscan Lux microplate reader (Thermo Fisher Scientific, Waltham, MA, USA). Readings were made at a wavelength of 450 nm. The reference wavelength was 650 nm.

### 2.5. Lactate Dehydrogenase Release Test

In order to finally confirm the effect of the tested flavonols on normal and cancer cells, the lactate dehydrogenase release test was performed. LDH is a stable cytosolic enzyme that is released only after cell membrane damage following necrosis. LDH activity was determined using a cytotoxicity assay kit (Cayman Chemical, Ann Arbor, MI, USA). Cells were treated with selected concentrations of flavonols for a specified period. The LDH released in the culture supernatants was detected via a coupled enzyme assay which converted the tetrazolium salt to a red formazan product. The absorbance was measured at 490 nm. Maximum LDH release control was performed by treating the cells with 1% Triton X-100 (Sigma Chemical Co.) for 10 min at room temperature.

### 2.6. Evaluation of Cell Morphology via H&E Cytological Staining

Cells were grown in 4-chamber microscopic culture dishes (Lab-Tek™, Waltham, MA, USA) at 5000 cells per well for 24 h. Then, the medium was exchanged for a medium with the addition of the tested flavonols in specific concentrations and left for 24, 48, and 72 h, respectively. After the specified time, the cells were fixed in 96% ethanol for 12 h and then dehydrated in a series of decreasing concentrations of ethanol, i.e., 99.6%, 96%, 90%, 80%, 70%, and 50%. The slides were rinsed with PBS (30 s) and immersed in hematoxylin for 6–8 min. The cells were then washed under running water (20 min) and stained with eosin for 30 s. After staining, the cells were washed again with PBS solution and dehydrated using increasing concentrations of ethanol, i.e., 50%, 70%, 80%, 90%, 96 %, and 99.6%. Finally, the slides were immersed in an ethanol/xylene mixture (50% vs. 50%) and then in pure xylene and sealed. The slides stained in this way were analyzed under the Olympus BX41 microscope (Tokyo, Japan) [32,33].

### 2.7. Evaluation of Apoptosis and Necrosis Using Fluorescence Microscopy

The analysis was performed using the Apoptotic, Necrotic, and Healthy Cells Quantification Kit (Biotium, Hayward, CA, USA). For this purpose, cells were cultured in 4-chamber culture dishes at 5000 cells per well for 24 h. Then, the medium was exchanged for a medium with the addition of the tested flavonols in specific concentrations and left for 24, 48, and 72 h, respectively. After this time, the cells were rinsed twice with PBS and a previously prepared staining solution containing Annexin V -FITC, 5 EthD-III, and Hoechst 33342 in binding buffer was added. Cells were incubated with dyes for 15 min at room temperature and protected from light. Cells were washed twice with binding buffer and analyzed using a Leica DMi8 fluorescence microscope (Mannheim, Germany).

### 2.8. Flow Cytometry Analysis

#### 2.8.1. FITC Annexin V Apoptosis Detection Kit II

Cells were seeded into culture dishes at a density of 5 × 10^5^ cells/dish and incubated for 24 h at 37 °C and 5% CO_2_. After incubation, cells were stripped and then washed twice with PBS. The apoptotic effects of the flavonols were analyzed using the Annexin V Apoptosis Detection Kit II (BD Pharmingen, San Diego, CA, USA), which contains Annexin V binding buffer -FITC and PI staining buffer.

Briefly, cells were suspended in 100 μL of binding buffer and sequentially mixed with 5 μL of Annexin V-FITC and 5 μL of PI. The mixture was incubated for 15 min at room temperature in the dark. Cell apoptosis was quantified using a CytoFlex Srt flow cytometer (Beckman Coulter, Miami, FL, USA) and dedicated CytExpert Srt software Version 1.1.

#### 2.8.2. Cell Cycle Analysis—PI/RNase Staining Buffer

The initial stages of sample preparation were analogous to the above. After washing the cells twice with PBS, the cells were centrifuged at 1000 rpm for 10 min and then the supernatant was aspirated. The cells were vortexed (to loosen the pellet) by dropwise addition of 5 mL of cold 70% ethanol and incubated at −20 °C overnight. After this time, the cells were washed twice to remove the ethanol. The cells were then centrifuged for 10 min at 1000 rpm and the supernatant was aspirated. For cell staining, 0.5 mL of PI/RNase staining buffer (BD Pharmingen, San Diego, CA, USA) containing propidium iodide and RNase was added to each sample. The cells were incubated with the dye for 15 min at RT. Samples were stored at 4 °C and protected from light until analysis. Stained cells were analyzed on a CytoFlex Srt flow cytometer to determine the relative DNA content, which was based on red fluorescence intensity.

#### 2.8.3. Staining Cells with JC-1

Initial sample preparation steps were analogous to those for the Apoptosis Detection Kit II assay. At the end of the flavonol treatment period, the cells were trypsinized and transferred to a sterile 15 mL polystyrene tube. The cells were then centrifuged at 400× *g* for 5 min at room temperature and the supernatant was removed. An amount of 0.5 mL of freshly prepared JC-1 working solution in which the cells were suspended was added to each sample. Cells were incubated for 15 min at 37 °C. After incubation, the cells were washed twice. After washings, each cell pellet was suspended in 0.5 mL of 1x assay buffer and vortexed. The analysis was performed via excitation of the signal with a laser with a wavelength of 488 nm. Readings were made at 525 nm and 590 nm on a Cytoflex Srt cytometer.

#### 2.8.4. PE Active Caspase-3 Apoptosis Kit

The initial stages of sample preparation were analogous to the previous tests. The cells were then washed twice with cold 1X PBS and suspended in BD Cytofix/Cytoperm™ solution at a concentration of 1 × 10^6^ cells/0.5 mL and the cells were incubated for 20 min on ice. The cells were then centrifuged to remove the BD Cytofix/Cytoperm™ solution and washed twice with BD Perm/Wash™ buffer (1X) at 0.5 mL buffer/1 × 10^6^ cells. Cells were then suspended in BD Perm/Wash™ (1X) buffer with antibodies and incubated for 30 min at room temperature. After this time, each sample was washed with 1.0 mL of BD Perm/Wash™ buffer (1X) and then resuspended in 0.5 mL of BD Perm/Wash™ buffer (1X) and analyzed on a BD Accuri C6 with the BD Accuri Software Version 1.0.23.1 (BD Biosciences, San Jose, CA, USA).

#### 2.8.5. PE Anti-Human Bcl-2 Kit

The same methodology applied for the PE Active Caspase-3 Apoptosis Kit was used to determine the Bcl-2 protein. The intracellular staining procedure was used. Intracellular labeling requires cell fixation and permeabilization. For this purpose, the BD Cytofix/Cytoperm™ Fixation/Permeabilization Kit (Cat. No. 554714) was used and all steps were performed according to the manufacturer’s instructions. Following the procedure, intracellular staining was performed using an anti-Bcl-2 antibody labeled with PE. In addition, an IgG isotype control was prepared using the same dilutions as the primary antibodies. Cells were analyzed using a BD Accuri C6 cytometer with the BD Accuri Software (BD Biosciences, San Jose, CA, USA).

### 2.9. Cell Migration Test

Cells were seeded in 6-well plates and allowed to expand to 90% confluence before wound formation using a 200 µL pipette tip. The cells were washed with sterile PBS to remove residues before being treated with ½ IC_50_ flavonols or DMSO solvent for 24 h. Analysis was performed using the Juli™ Br culture cell observation system (NanoEntek, Seoul, Republic of Korea). The results were analyzed using CorelDraw 22 software (Corel Corporation, Ottawa, ON, Canada) and expressed as the percentage of area covered by cells.

### 2.10. Cytochrome c Release Assay

To determine cytochrome c release, cells were plated in 6-well plates at 4 × 10^5^ cells/well and left for 24 h. After this time, the medium was added to the wells along with the appropriate concentration of the test compound. Cells were harvested 6 h later, and then washed and centrifuged. The cell pellets were then lysed and centrifuged again. Cytosolic cytochrome c levels were measured with the Cytochrome c ELISA kit (Human Cytochrome C ELISA Kit Diaclone, Besançon, France) and performed according to the manufacturer’s instructions. Readings were made at a wavelength of 450 nm. Cytochrome c cytosol levels are expressed as a percentage of control.

### 2.11. Statistical Analysis

Statistical significance of differences was calculated using Student’s t-test for comparing data between two groups and one-way ANOVA for comparing data from more than two groups. Statistica software (Ver. 13.1, StatSoft, TIBCO Software, Palo Alto, CA, USA) was used for data analysis. *p* < 0.05 was considered statistically significant.

## 3. Results

### 3.1. Kaempferol and Fisetin Inhibit HNCs Cell Proliferation

**Figure 1:**
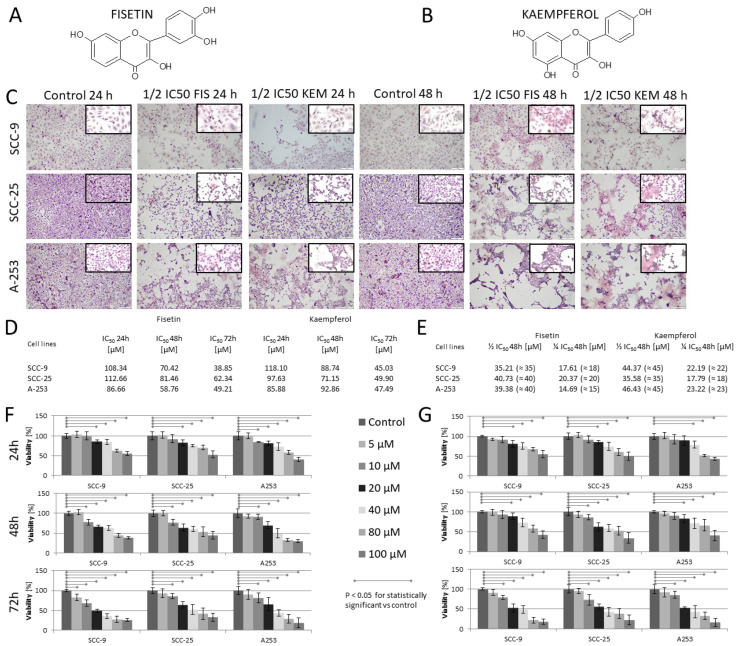
Cytotoxic effect of fisetin and kaempferol on SCC-9, SCC-25, and A-253 head and neck cancer cells. Chemical structure of fisetin (**A**) and kaempferol (**B**). (**C**) Representative images of hematoxylin and eosin staining (low (100×) and high (400×) optical magnification images). (**D**) The calculated IC_50_ values of fisetin and kaempferol for 24-, 48-, and 72-h cell incubations are presented in the table. (**E**) The calculated ½ and ¼ IC_50_ values were used for further research. (**F**) Visual graphs presenting the dose- and time-dependent decrease in cell viability of the test lines after treatment with fisetin (0–100 µM) for 24, 48, and 72 h, respectively. Fisetin showed no significant difference in Beas-2B cell viability (normal cell line). All cells were compared with DMSO controls. The IC_50_ of fisetin after 72 h of incubation was found to be the lowest for the SCC-9 line and the highest for the SCC-25 line. (**G**) The figure shows the decrease in viability of cells treated with kaempferol depending on the time and dose, and the IC_50_ values ranged from 45.03 to 49.90 µM. No statistically significant differences in kaempferol IC_50_ values after 72 h between cell lines were found.

To test the antiproliferative and cytotoxic activity of kaempferol and fisetin on HNC cells (Figure 1), cells were treated with various concentrations of kaempferol and fisetin for 24, 48, and 72 h and subjected to LDH and WST-1 assays. The WST-1 assay demonstrated that cell proliferation was inhibited by both substances in a dose-dependent manner, with IC_50_ values of 38.85 µM, 62.34 µM, and 49.21 µM for fisetin and 45.03 µM, 49.90 µM, and 47.49 µM for kaempferol (Figure 1D), respectively, for lines SCC-9, SCC-25, and A-253 after 72 h of incubation. Then, tests were performed on control cells, i.e., normal epithelial cells of the Beas-2B line, using only ½ IC_50_ and ¼ IC_50_ (for fisetin and kaempferol) concentrations for cytotoxicity assessment, as these concentrations were used for further studies. The control line and the concentrations analyzed did not show a cytotoxic effect above 5% toward Beas-2B cells in any of the tests performed.

### 3.2. Kaempferol and Fisetin Induce Cell Cycle Arrest in SCC-25, SCC-9, and A-253 HNC Cell Lines

**Figure 2:**
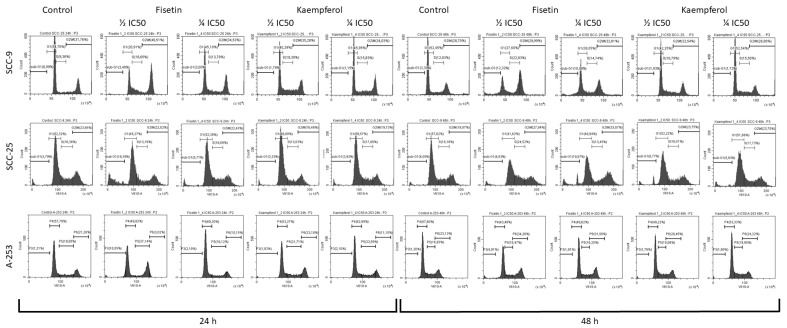
Cell cycle analysis via flow cytometry: Analysis of the DNA content of fisetin- and kaempferol-treated cells for 24 and 48 h was compared with DMSO control cells after PI staining. Cells in the S phase had more DNA than cells in the G1 phase, so they absorbed proportionally more dye until their DNA content doubled. The signal from cells in the G2 phase is roughly twice as bright as that from cells in the G1 phase. The distribution of PI-stained DNA content also allows the detection of apoptotic cells with fractionated DNA content. The performed analysis showed cell cycle arrest in the G2/M phase with an increase in the sub-G1 apoptotic population in SCC-9 cells treated with fisetin for 48 h. For the SCC-25 line, a significant increase in apoptotic cells in the sub-G1 phase was observed after 24 h of incubation with fisetin at a concentration of ½ IC50. The inhibition of the cycle in the G1 phase was observed when cells were treated with kaempferol. In the case of the A-253 line, cells in the sub-G1 phase were not observed and the effect of fisetin usually resulted in the inhibition of the cell cycle in the G2/M phase. It has also been shown that kaempferol may promote cell inhibition by inducing S-phase cell cycle arrest in the SCC-9 cell line.

Since treatment with kaempferol and fisetin inhibited the proliferation of tongue and salivary gland cancer cells, we investigated whether this was due to the induction of cell cycle arrest. The role of the tested flavonols in cell cycle arrest was analyzed using flow cytometry using a cell cycle phase detection kit (Figure 2). The data indicate that fisetin decreased the cell population in the G1 phase and adequately increased the cell population in the G2/M phase. An increase in cells in the sub-G1 phase was also noted after treatment with fisetin, particularly in cells of the SCC-25 line. After treating SCC-9 cells with kaempferol for 24 h, the population of G1 cells was significantly reduced from 54% to 40%, and the population of S-phase cells was significantly increased from 9 to 19%, indicating that kaempferol contributed to stopping the cycle in the S phase. The results showed that kaempferol promotes cell inhibition by inducing cell cycle arrest. For the A-253 line, we observed inhibition of the cell cycle in the G2/M phase because of the cell’s exposure to fisetin at a concentration of ½ and ¼ IC_50_.

### 3.3. Kaempferol and Fisetin Induce Apoptosis in HNC Cells

**Figure 3:**
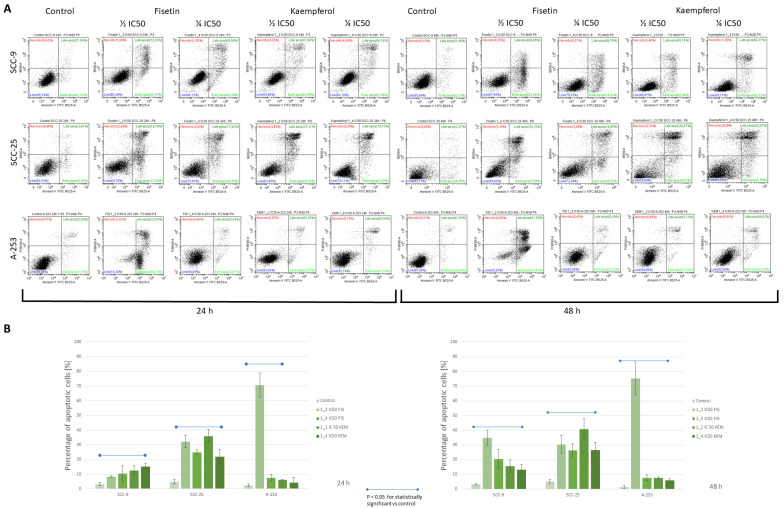
Annexin V assay result. To facilitate the differentiation of necrotic and apoptotic cells, the cells were additionally stained with a solution of propidium iodide (PI). Lower left quadrant (FITC-/PI−) = viable cells, lower right quadrant (FITC+/PI−) = early apoptotic cells, upper right quadrant (FITC+/PI+) = late apoptotic cells, and upper left (FITC-/PI+) = necrotic cells. (**A**)—Representative scatter plots of PI (y axis) vs. annexin V (x axis). The variable, consistent influence of fisetin and kaempferol on the induction of apoptosis has been noted. After 48 h of incubation of cells with fisetin at a concentration of ½ IC_50_, the percentage of Annexin -V (+) cells increased to about 75% and 30%, for A-253 and SCC-25/SCC-9 lines, respectively. An evident effect of kaempferol on SCC-25 cells was also observed, including the elevation of Annexin-V (+) cells to about 37%. (**B**)—Percentage of apoptotic cells (early + late). Data are presented as the means ± SE of triplicate experiments. *p* < 0.05 vs. control.

Since kaempferol and fisetin showed cytotoxic activity for the investigated cancer cells, we assessed whether this was related to the induction of apoptosis. Our study revealed that exposure to kaempferol and fisetin induces apoptosis in a time- and dose-dependent manner (Figure 3). In addition, treatment of the cells with kaempferol enhanced the Annexin V (+) cell population in all tested cells. However, the strongest effect was observed when fisetin was used at a concentration of ½ IC_50_ against A-253 cells. In A-253 salivary gland cancer cells, a slight effect of kaempferol on the activation of the apoptosis process was observed. A more potent effect of kaempferol was observed in the SCC-9 and SCC-25 tongue cancer lines, where the percentage of Annexin V (+) cells increased to 35%. While the induction of apoptosis followed the application of the tested compounds, subsequent evaluation was performed to verify a caspases-related pathway.

### 3.4. Kaempferol and Fisetin Change the Potential of the Mitochondrial Membrane

**Figure 4:**
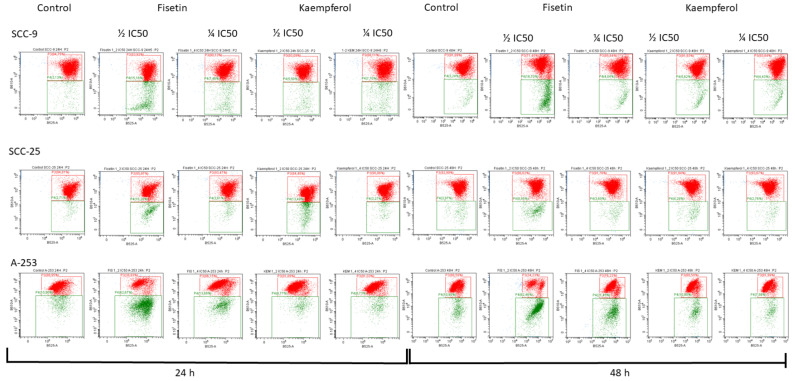
Flow cytometry graph demonstrating the gating of the population of JC1 aggregates (red) and JC1 monomers (green). JC-1 staining shows decreased mitochondrial membrane potential (Ψm) in tongue and salivary gland cancer cells treated with fisetin and kaempferol for 24 and 48 h.

Since treatment with kaempferol and fisetin induced apoptosis of tongue and salivary gland cancer cells, we examined the impact of tested flavonoids on alterations in the potential of mitochondrial membranes. Scientific evidence suggests that mitochondria play a key role in apoptosis by releasing cytochrome c and other proteins that are necessary to activate pro-caspase-9 and carry out apoptosis. As a result, caspase-9 activated by the mitochondria can activate caspase-3. The obtained results showed a decrease in the potential of the mitochondrial membrane in cells treated with fisetin and kaempferol (Figure 4). In comparison, the greatest diminishment in potential was observed in the A-253-line cells exposed to fisetin. In comparison, significant changes in potential were also observed in the SCC-9 and SCC-25 lines. Interestingly, kaempferol did not cause a significant disturbance in the potential value, and this observation is consistent with the results obtained from apoptosis assay.

### 3.5. Kaempferol and Fisetin Induce Apoptosis in HNC Cells by Activating Caspase-3

**Figure 5:**
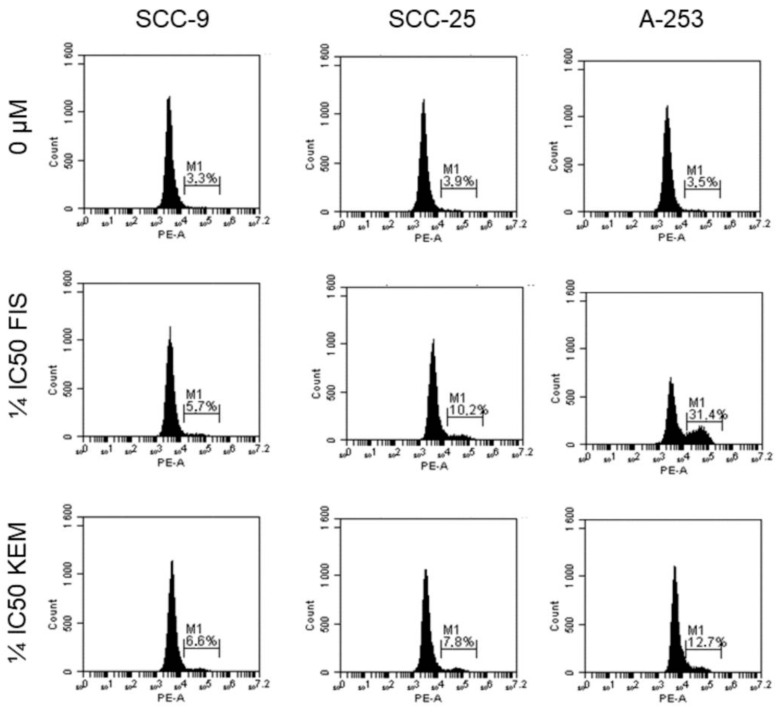
Detection of apoptosis via flow cytometry after staining with anti-caspase-3 antibody. Active caspase-3 proteolytically cleaves and activates other caspases. SCC-9, SCC-25, and A-253 cells were treated with fisetin or kaempferol for 48 h. (Viable cells are in the left quadrant, while the presence of active caspase-3 is shown in the right quadrant (M1)). Caspase-3 activity in A-253 cells was significantly increased in cells treated with kaempferol and fisetin compared with untreated cells. The lesser caspase-3 activity was observed in the SCC-9 line (the increase in caspase-3 was not statistically significant).

Since treatment with kaempferol and fisetin induce apoptosis of tongue and salivary gland cancer cells, we investigated whether this activation was mediated by caspase-3. In the execution phase, substrates are cleaved under the influence of effector caspases, including caspase-3, e.g., cytokeratin, PARP, and cytoskeleton membrane proteins. As the main executive role is played by caspase-3, which may be activated in various ways by caspase-8, -9, and -10, flow cytometry was employed to investigate whether caspase-3 is activated in cells exposed to kaempferol and fisetin. Arbitrarily, a lower concentration of kaempferol and fisetin was selected to minimize the impact of the flavonols’ cytotoxicity on the obtained results. As shown in Figure 5, after treating the cells with fisetin at a concentration of ¼ IC_50_, an elevation in caspase-3 from 3.3% to 5.7%, 3.9% to 10.2%, and 3.5% to 31.4% was observed for SCC-9, SCC-25, and A-253 cells, respectively. Thus, the strongest activation of caspase-3 was observed with line A-253. A similar relationship was observed with kaempferol; however, in this case, the activation of caspase-3 was recorded to a lesser extent and amounted to 12.7% (A-253 line). The results firmly correlated with those obtained from flow cytometry (Annexin-V staining).

### 3.6. Kaempferol and Fisetin Inhibit the Anti-Apoptotic Proteins of the Bcl-2 Family

**Figure 6:**
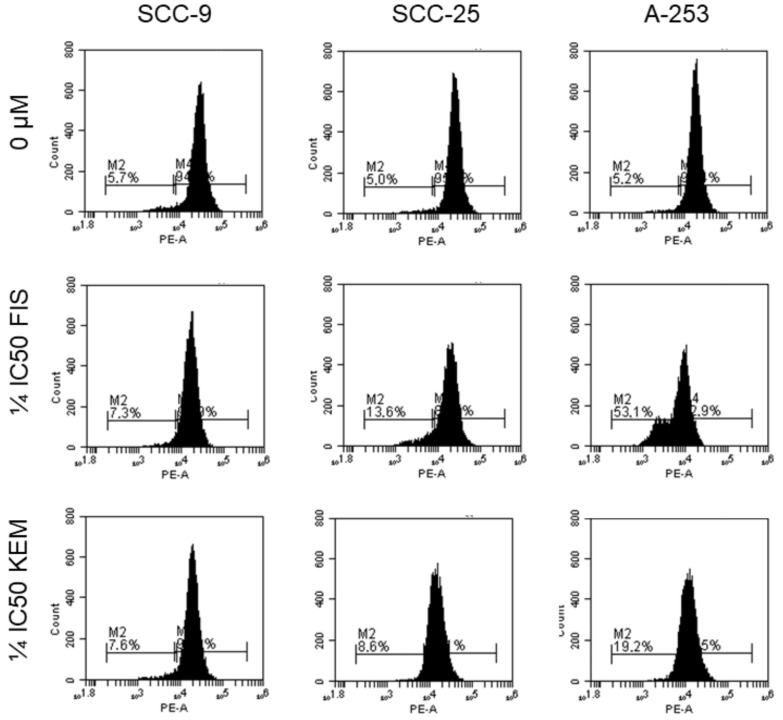
Detection of apoptosis via flow cytometry after staining with anti-Bcl-2 antibody. Bcl-2 is believed to provide cells with a selective survival advantage by blocking apoptosis and thus contributing to tumorigenesis. The effect of fisetin and kaempferol on the activity of the Bcl-2 protein is evident. These changes were particularly noticeable when fisetin was used on A-253 cells, revealing that such alterations were consistent with both the activation of caspase-3 and the percentage of Annexin-V (+) cells. For the A-253 line, the percentage of Bcl-2 negative cells increased from 5.2% to 53.1% for fisetin at the concentration tested and to 19.2% for kaempferol. No such significant changes were observed in the SCC-9 and SCC-25 lines; however, a similar elevation of Bcl-2-negative cells was noted following the use of flavonols. SCC-9, SCC-25, and A-253 cells were treated with fisetin or kaempferol for 48 h.

The evaluation of the anti-apoptotic protein Bcl-2 preventing cell death was carried out as this protein is an oncogene resulting from the translocation of chromosomes causing neoplastic transformation. Bcl-2 affects the permeability of the mitochondrial outer membrane, allowing the release of cytochrome c from the mitochondrial intermembrane space into the cytosol. Overexpression of the Bcl-2 protein is inherent in various cancers, including HNCs [34]. As shown in Figure 6, the percentage of cells with a strong Bcl-2 signal was present in all cell lines tested. After the use of kaempferol and fisetin, a weaker signal for the Bcl-2 protein and the appearance of Bcl-2 negative cells were observed. This suggests that the tested flavonols affect the expression of the anti-apoptotic protein Bcl-2.

### 3.7. Fisetin Increases the Release of Cytochrome c in Head and Neck Cancer Cells

**Figure 7:**
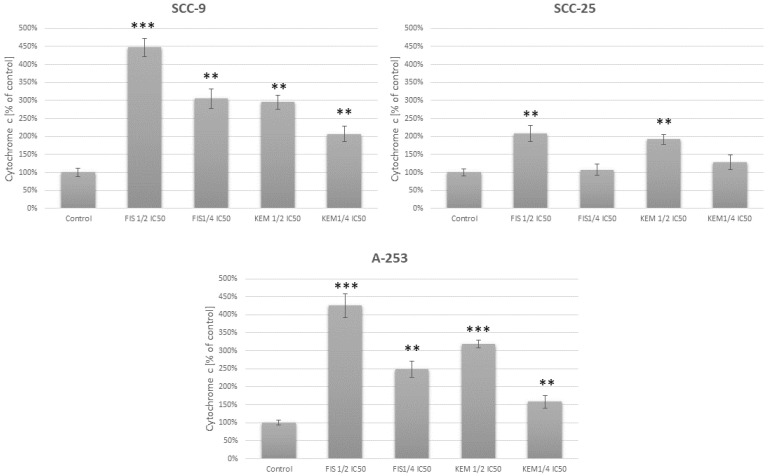
Effect of fisetin and kaempferol at ½ IC_50_ and ¼ IC_50_ concentrations on the cytosolic fraction of cytochrome c in SCC-9 and SCC-25 tongue cancer cells and A-253 salivary gland cancer cells. Fisetin at a concentration of ½ IC50 causes a significant release of cytochrome c from the mitochondria into the cytosol in all tested cells. A marked elevation in the level of cytochrome c was observed in SCC-9 and A-253 lines. Higher concentrations of fisetin and kaempferol do not cause statistically significant changes in the level of cytochrome c (SCC-25 line). ** *p* < 0.01; *** *p* < 0.001 compared with controls.

Cytochrome c is a component necessary for the key stages of apoptosis, i.e., caspase-3 activation and DNA fragmentation. Cytochrome c moves from the mitochondria to the cytosol during apoptosis while still in intact cells, and a decrease in the transmembrane potential of the mitochondria accompanies early apoptosis. The release of cytochrome c into the cytosol leads to the activation of apoptosis by activating the caspase-dependent pathway [35]. Cytochrome c achieves this goal by interacting with other cytosolic factors to form the apoptosome. The measurement of cytochrome c release from mitochondria is a tool to detect the first early stages of apoptosis initiation in cells. As the release of cytochrome c in the cytosol takes place before the activation of caspases and DNA fragmentation, we determined the level of cytochrome c in the 6th hour of cell incubation with the tested flavonols. As a result, we showed that fisetin and kaempferol influence the level of cytochrome c (Figure 7). Fisetin has the most potent effect on cells of the SCC-9 and A-253 lines, which correlates with the other results. Significantly less obvious results were obtained while incubating the SCC-25 line with both kaempferol and fisetin. Using the concentration of ¼ IC_50_, these results were not statistically significant.

### 3.8. Kaempferol and Fisetin Cause Morphological Changes in HNC Cells

As fisetin and kaempferol cause apoptosis of the tested cell lines, the morphology of cells treated with fisetin and kaempferol was evaluated using light microscopy. H&E staining demonstrated that fisetin- and kaempferol-treated cells revealed nuclear condensation and fragmentation, which was augmented with both concentration and time-point modes (Figure 1C). Additionally, we found alterations in the structure, size, and shape of the nuclei in HNC cells treated for 48 h, with large cytoplasmic vacuoles, and abnormal mitotic figures.

### 3.9. Kaempferol and Fisetin Induce the Formation of Apoptotic Bodies in HNC Cells

**Figure 8:**
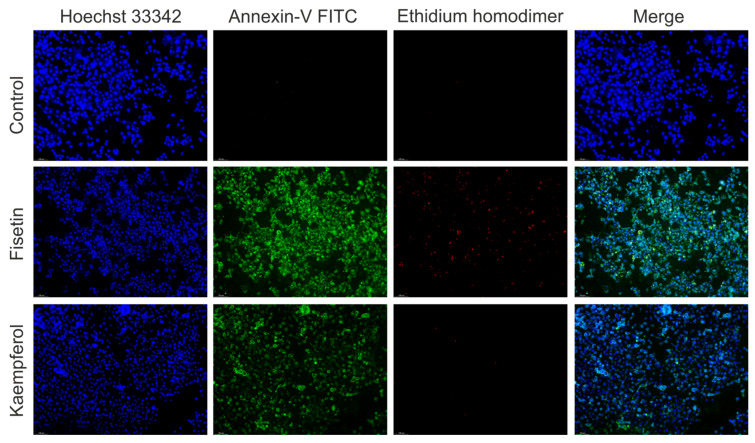
The representative microphotographs of A-253 cells stained with the Apoptotic, Necrotic, and Healthy Cells Quantification Kit using Hoechst 33342, Annexin-V FITC, and Ethidium homodimer III dyes. Apparent apoptotic changes resulting from 48 h of incubation of salivary gland cancer cells with fisetin and kaempferol at a concentration of ½ IC_50_. The cell nucleus was visualized with a blue signal, phosphatidylserine was visualized with a green signal, and necrotic cells were stained with a red signal. Optical magnification ×100.

As we highlighted, fisetin and kaempferol are responsible for the increase in the percentage of cells with active caspase-3. Caspase-3 causes cell disintegration and the formation of apoptotic bodies on the surface of which there is phosphatidylserine. Therefore, we checked using fluorescence microscopy whether apoptotic bodies are formed in cells and phosphatidylserine is translocated from the inner membrane to the outer membrane. The apoptotic cell usually separates from other cells. As a result of the loss of intracellular water and electrolytes, it shrinks and changes the shape, size, and density of the cytoplasm. The surface of such a cell is characteristically corrugated. At the same time, there are changes in the nuclear chromatin (Figure 8). It thickens and initially accumulates near the nuclear membrane. It then fills the entire nucleus, which becomes pyknotic. The organelles are densely packed in the cytoplasm and show no significant morphological changes. A characteristic feature of apoptosis is also the fragmentation of the cell nucleus and the formation of the so-called apoptotic bodies in the later stages of this process.

### 3.10. Kaempferol and Fisetin Inhibit the Migration of HNCs Cells

**Figure 9:**
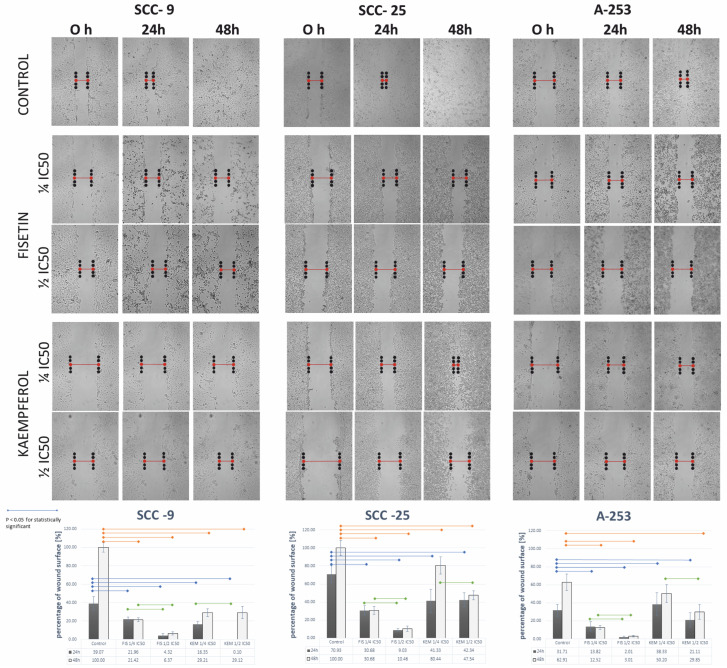
The representative microphotographs of SCC-9, SCC-25, and A-253 cells were incubated for 24 and 48 h with fisetin and kaempferol. Fisetin and kaempferol inhibit wound healing in 2D cultures of the SCC-9, SCC-25, and A-253 lines. The activity of fisetin and kaempferol was tested after 24 and 48 h of cell incubation and a significant dose-dependent decrease in wound healing areas was observed compared with the untreated control. The data expressed in the bar graphs represent the average scratch area values in percent compared with the 0 h control and the 24 h and 48 h controls from three independent experiments.

As may be seen in the SCC-9 and SCC-25 lines, cells untreated with flavonols became overgrown in the entire free space after 48 h. The exposure to fisetin and kaempferol inhibited the motility of HNC cells in a dose-dependent manner. A wound-healing assay was performed using cells of the SCC-9, SCC-25, and A-253 lines treated with fisetin and kaempferol. As shown in Figure 9, cells of the above-mentioned lines treated with fisetin and kaempferol migrated more slowly than cells in the control group after 24 and 48 h of incubation. In the case of the SCC-9 line, cells treated with flavonols were only overgrown in the free space at a maximum of 30%, and the inhibition of cell motility of this line was the strongest.

## 4. Discussion

Knowledge about the impact of flavonols on head and neck cancer cells is insufficient despite the growing research evidence. Several publications exist confirming that flavonols induce apoptosis of human cells, particularly in oral cancer. Potential anti-cancer properties exhibited by fisetin and kaempferol include antiproliferation, tumor growth arrest through modulation of key cell cycle regulators, and apoptosis. Apoptosis-inducing properties in transformed cells are key features of a potential chemopreventive agent [36]. Numerous reports have shown that fisetin and kaempferol induce cytotoxic effects on human cell lines by arresting the cell cycle and apoptosis, such as: bladder cancer [36], intestinal cancer [27,37], breast cancer [38,39], gastric cancer [40,41], prostate cancer [42], pancreatic cancer [43], ovarian cancer [44], liver cancer [45], skin cancer [46], cervical cancer [36], lung cancer [47], brain cancer [48], osteosarcoma [49], melanoma [50], multiple myeloma [51], leukemia [18,52], endometrial cancer [53], and head and neck cancers [16].

**Figure 10:**
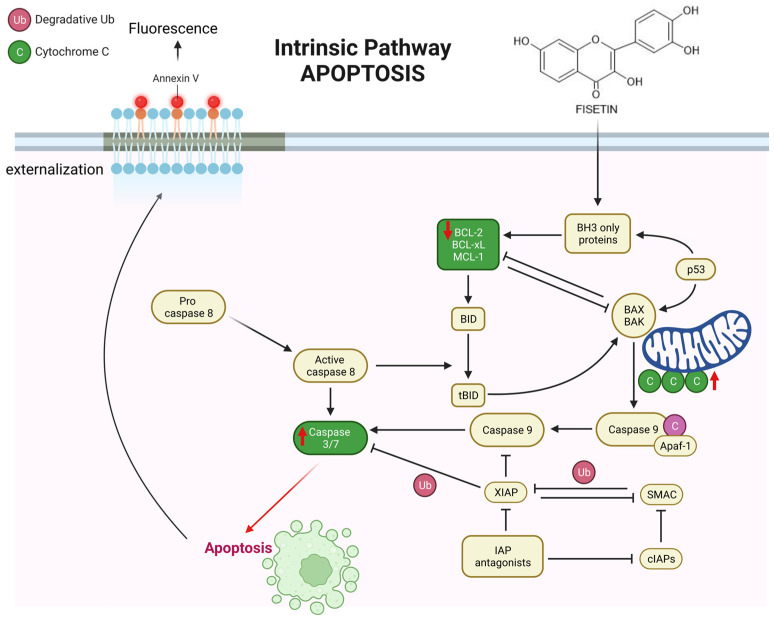
The molecular elements of apoptotic cell death in intrinsic pathway activation by fisetin. The figure shows the importance of the regulatory role of proteins belonging to the Bcl-2 protein family in the apoptosis process. The anti-apoptotic proteins group: Bcl-2, Bcl-XL, Bcl-w, and Mcl-1, as well as pro-apoptotic proteins: Bax, Bak and Bik, Bad, Bid, Bim, NOXA, and PUMA. Pro-apoptotic proteins induce pores in the mitochondrial membrane, with a subsequent release of various apoptotic factors from the mitochondria into the cytosol. In turn, the release of these molecules can be inhibited by anti-apoptotic proteins. The release of cytochrome c is the signal for the formation of the apoptosome, which begins with the binding of cytochrome c to the Apaf-1 protein, ATP, and procaspase-9. Subsequently, procaspase-9 activates the effector caspase 3. The apoptosome thus activates the caspase cascade, which ultimately leads to cell destruction (short, red upward arrows symbolize the up-regulated protein content, while short, red downward arrows symbolize the down-regulated protein content; created using BioRender platform).

Knowledge about the impact of flavonols on head and vision cancer is insufficient. In the available literature, a publication confirmed that flavonols induce apoptosis of human cancer cells, particularly in oral cancer; however, the mechanisms of activation have not been fully understood (Figure 10).

Our comprehensive, multifaceted study investigated the cytotoxic effects of fisetin and kaempferol on human tongue and salivary gland cancer cells in vitro. We confirmed that fisetin inhibits proliferation and acts as an inducer of apoptosis in SCC-9, SCC-25, and A-253 cells. Moreover, the results revealed that flavonols reduce cell viability in both a concentration- and time-dependent manner, with IC_50_ values of 38.85 µM; 62.34 µM and 49.21 µM for fisetin and 45.03 µM; 49.90 µM and 47.49 µM for kaempferol after 72 h for the SCC-9, SCC-25, and A-253 lines, respectively. Compared with the other studies, the IC_50_ of fisetin was amounted at 40 µM for HSC3 cells [54], 52,8 µM for SCC-4 cells [23], 20 µM for HSC3 cells [55], 50 µM for Cal-27 cells [56], and about 10 μM for Hep-2, Tu212, and M2e cells [57]. To date, the effect of fisetin on SCC-9 and SCC-25 tongue cancer cells and A-253 salivary gland cancer cells was not distinctly investigated. In the case of kaempferol, the IC_50_ values were also consistent with the results obtained by our team, with the SCC-1483 line having an IC_50_ value of 40 μM [58] and 45 μM for the FaDu line [29]. Other reports suggested that kaempferol at a concentration of up to 100 μM does not affect the viability of SCC-4 tongue cancer cells; however, it inhibits the migration and invasion of cancer cells [59]. Contrarily, our results indicated that kaempferol is cytotoxic to tongue and salivary gland cancer cells.

To further investigate whether the tested flavonols arrest the cell cycle and induce cell death by apoptosis in cells, a panel of comprehensive assays was utilized to validate findings. Our study evidently confirmed that fisetin reduces the population of cells in the G1 phase and increases the population of cells in the G2/M phase accordingly. Growth of cells in the sub-G1 phase was also observed after fisetin treatment of the cells. It was also shown that kaempferol contributed to the arrest of the cell cycle in the S phase. Interestingly, the arrest of cells in the S phase of the cell cycle may be a transient process, and the compounds themselves may act as reversible inhibitors of eukaryotic nuclear DNA replication, blocking the passage of cells beyond the S phase. Arguably, this mechanism may be related to the inhibition of DNA polymerase; however, the evaluation of this mechanism requires further research. A vast proportion of results obtained in other studies correlate with our results and indicate that fisetin induces G2/M phase arrest and sub-G1 phase induction [23]. Kaempferol has also been shown to arrest the cell cycle in the G2/M phase in HT-29 colon cancer cells [60], A2780/CP70 ovarian cancer cells [61], 786-O and 769-P kidney cancer cells [62], and breast cancer of the MDA-MB-453 line [63].

Fisetin-induced apoptosis of human oral cancer cells may occur, among others, through the generation of reactive oxygen species and mitochondria-dependent signaling pathways [23]. Dysregulation of apoptosis is essential for tumor formation and progression. To thoroughly study the process of apoptosis, we used several methods, including fluorescence and cytometric staining with Annexin-V, cytometric evaluation of active caspase-3, and evaluation of Bcl-2 protein expression. In this study, we showed that in cells treated with fisetin and kaempferol, the externalization of phosphatidylserine occurs, which is one of the first signs of activation of the apoptosis process, and is associated with a strong signal from the cytoplasm of the examined cells. The strongest signal was observed with fisetin-treated A-253 cells. In addition, we confirmed that the percentage of Annexin-V (+) cells increased in a dose-dependent manner and cell incubation time. Studies performed by other research teams showed strong activation of the apoptosis process dependent on the concentration of fisetin in the oral squamous cell cancer cells of the UM-SCC-23 line [64].

In evaluating the results of other studies, treatment with fisetin activates the proteins Bax and Bak, inhibits Bcl-2 and Bcl-XL, and increases the permeability of the mitochondrial membrane in CAL-27 and Ca9-22 cells. Moreover, it was confirmed that treatment of cancer cells with fisetin forms activated and cleaved caspase-3 and PARP. This finding suggests that fisetin treatment induces apoptosis via an intrinsic pathway and caspase activation, especially in the case of CAL-27 cells, which were more sensitive to fisetin treatment than Ca9-22 cells [56]. As our studies have shown, a decrease in the level of the anti-apoptotic protein Bcl-2 was observed, especially after the application of fisetin to A-253 salivary gland cancer cells. Caspase-3 levels also increased most strongly in line A-253 after treatment with both fisetin and kaempferol.

Noteworthy, mitochondria play an important role in stimulating apoptosis through a mechanism known as the intrinsic signaling pathway. Fisetin has been reported to interfere with the loss of Ψm leading to apoptosis and inhibition of tumor growth, accumulation of excess ROS causing nuclear DNA damage, disruption of Ψm, and release of cytochrome c into the cytosol. As demonstrated in previous laboratory-based studies, fisetin radically reduces the potential of the mitochondrial membrane in the CAL-27 and Ca9-22 cells [56]. Here, we did not observe such major discrepancies in the potential of the mitochondrial membrane in the SCC-9 and SCC-25 lines. Contrarily, a strong decrease in membrane potential was observed in A-253 cells exposed to fisetin.

## 5. Conclusions

While biologically potent substances such as kaempferol and fisetin have specific favorable properties against cancer cells, they have attracted attention as promising compounds for innovative and effective cancer therapies. The cytotoxic effects of fisetin and kaempferol on human tongue and salivary gland cancer cells in vitro appear significant, dose-dependent, although variable, and based primarily on the reduction in cell viability by inducing apoptosis pathway. With a different magnitude, fisetin and kaempferol trigger apoptotic cell death by disrupting the membrane potential of the mitochondria, resulting in the release of cytochrome c from the mitochondria into the cytosol, and subsequent activation of caspase-3. Increasing scientific evidence suggests that flavonols may become precursors of safe and cost-effective anti-cancer drugs, used alone or as an adjunct of standard chemotherapy.

## Data Availability

Not applicable.
